# *Gardnerella* vaginalis, from the Vaginal Microbiota to Prosthetic Joint Infection

**DOI:** 10.7150/jbji.32471

**Published:** 2019-08-06

**Authors:** Marion Thomas, Valérie Zeller, Beate Heym, Vanina Meyssonnier, Simon Marmor, Jean-Marc Ziza

**Affiliations:** 1 Service de Médecine Interne et Rhumatologie, Groupe Hospitalier Diaconesses-Croix Saint-Simon, Paris, France; 2 Centre de Référence des Infections Ostéo-Articulaires Complexes, Groupe Hospitalier Diaconesses-Croix Saint-Simon, Paris, France; 3 Laboratoire des Centres de Santé et Hôpitaux d'Île-de-France, Groupe Hospitalier Diaconesses-Croix Saint-Simon, Paris, France; 4 Service de Chirurgie Osseuse et Traumatologique, Groupe Hospitalier Diaconesses-Croix Saint-Simon, Paris, France

**Keywords:** prosthetic joint infection, *Gardnerella vaginalis*, hematogenous infection, vaginosis

## Abstract

We describe two cases of chronic *Gardnerella vaginalis* prosthetic hip infections, in an immunocompetent postmenopausal woman and a young immunocompromised woman. *G. vaginalis* was also isolated from the genital tract, suggesting hematogenous spread of the bacterium. Outcomes were favorable after one-stage exchange arthroplasty and prolonged antibiotic therapy.

## Background

*Gardnerella vaginalis,* a small, facultative, anaerobic rod, is known to be associated with bacterial vaginosis (BV) 1-5. Recent studies 2-5 still question the etiology of BV and the precise role of *G. vaginalis* in this local infection. Extravaginal *G. vaginalis* infections are very infrequent. Rare bone-and-joint infections have been reported 6-10. Only one joint arthroplasty infection has been published to date 10.

We describe two prosthetic hip infections due to this bacterium, their diagnoses, treatments and outcomes.

## Case presentation

### Case 1

In October 2016, a 68-year-old woman consulted her doctor for right groin pain. Her medical history included depression and nonmetastatic melanoma treated by excision. She had given birth to three children, then 50, 48 and 35 years old. She had no prior abortion, or gynecologic or urologic procedures.

Eleven years ago, fracture of the right acetabulum was treated by osteosynthesis, followed 1 year later by total hip arthroplasty for posttraumatic osteoarthritis. She did well for 10 years.

**Figure 1:**
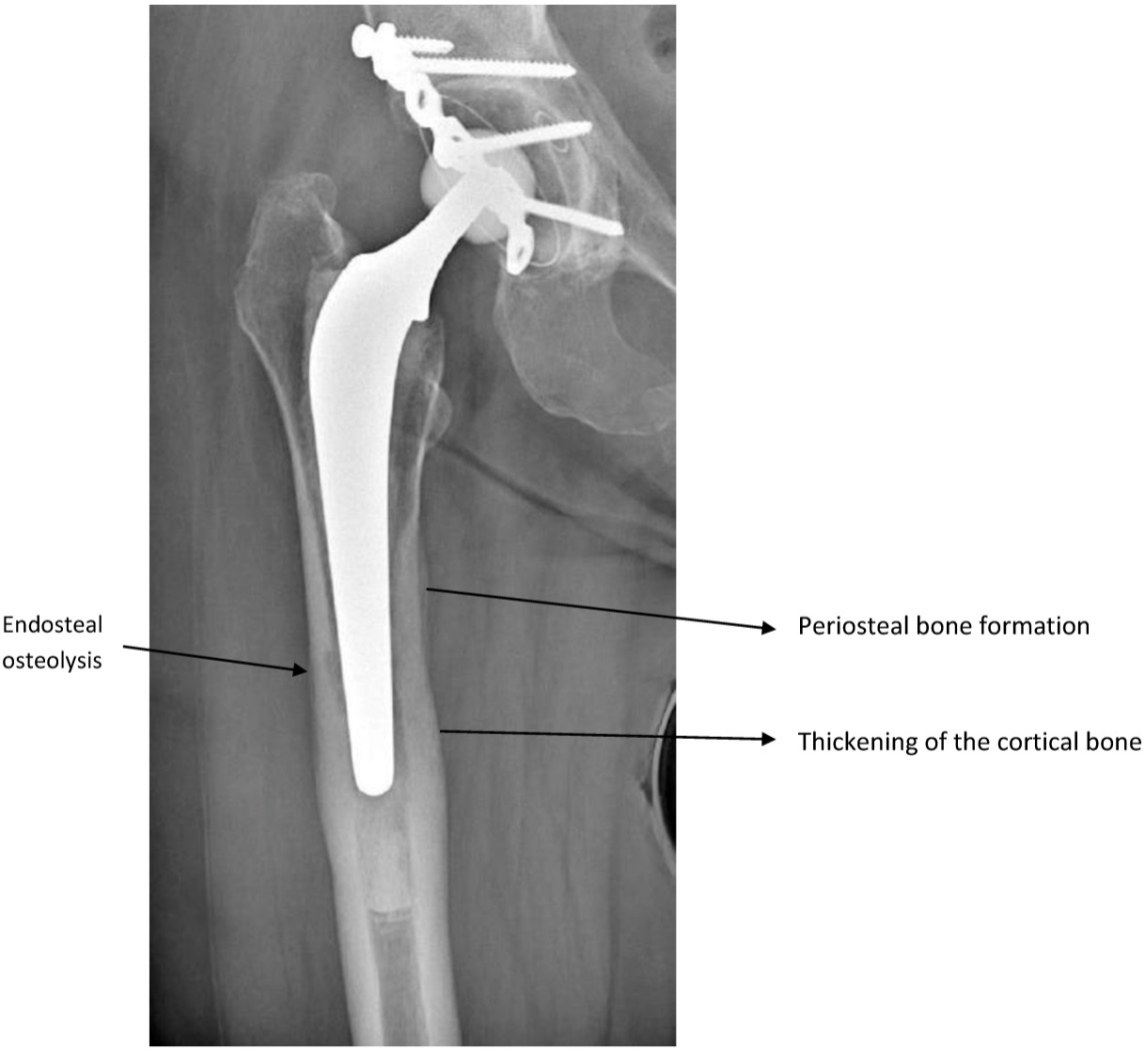
X-ray of Case 1's right hip showing a cemented hip arthroplasty with signs of chronic femoral infection: endosteal osteolysis, periosteal bone formation and thickening of the cortical bone.

Four months before her first visit in our center, she developed sudden mechanical pain in her right groin that rapidly became debilitating. She had no fever, sweating or rigors. Her C-reactive protein (CRP) concentration was 46 mg/L (normal <5 mg/L) and white blood-cell count normal. X-rays (Figure 1) showed signs of chronic femoral infection. Suspecting prosthetic joint infection (PJI), a hip aspirate was obtained. Joint-fluid white-cell count was 12.42 G/L, 96% neutrophils. Gram-stained smear and search for crystals were negative. Cultures on chocolate and Columbia blood agar (BioMérieux, Marcy l'Etoile, France), incubated in a CO_2_-enriched anaerobic atmosphere, yielded numerous small (<1-mm diameter), circular, convex, gray colonies with a prominent zone of beta-hemolysis on blood agar, identified as *G. vaginalis* by MALDI-TOF mass spectrometry (Bruker, Bremen, Germany; score 2.1). No other pathogen was isolated. Susceptibility testing, carried out by disk diffusion on Mueller-Hinton F medium under anaerobic conditions and analyzed according to the European Committee on Antimicrobial Susceptibility Testing 11, showed susceptibility to penicillin, amoxicillin (minimal inhibitory concentration (MIC) 0.064 mg/mL), clindamycin, rifampicin and metronidazole. This postmenopausal patient had no history of sexually transmitted diseases or clinical signs of vaginosis. Gynecologic examination was unremarkable, pelvic ultrasound discovered a myoma. A vaginal smear and swab culture confirmed the presence of *G. vaginalis* with clue cells, and absence of *Lactobacillus* and normal flora. Nugent score was 7. This score, the reference method for microscopic reading of vaginal smears, is one of the diagnostic methods for BV. It assesses vaginal microbial flora changes by counting *Lactobacillus*, *Mobiluncus* and *Gardnerella*-like bacteria 12. Searches for other pathogens (*Trichomonas vaginalis*, *Neisseria gonorrhoea*, *Chlamydia trachomatis*, *Candida* spp.), and serology for antibodies to *Treponema pallidum*, human immunodeficiency virus, hepatitis B and C viruses (COBAS^©^ 6000 analyzer; Roche Diagnostics SA, Industriestrasse 7, 6343 Rotkreuz, Switzerland) were negative. Urine culture was negative; no other samples (e.g., pharynx) were taken. Serum protein electrophoresis, immunoglobulins G, A and M, and complement levels were normal.

Six months after the pain started, she underwent one-stage exchange arthroplasty. During surgery, loosening of the acetabulum and mild periprosthetic tissue inflammation were observed, but not purulence. Cultures of four intraoperative samples confirmed *G. vaginalis* infection. The prosthesis was not sonicated. Treatment with continuous intravenous (IV) clindamycin (2400 mg/24h) was started preoperatively after samples were obtained. Ten days later, a diffuse maculopapular rash, suggestive of drug allergy, appeared. Clindamycin was switched to IV amoxicillin (12 g/24 h) for 18 days, followed by 8 weeks of oral amoxicillin (2 g, TID). Twenty-five months post-surgery she was doing well, with no pain. The scar was unremarkable and CRP normal. X-ray of the right hip showed no osteolysis around the prosthesis.

### Case 2

A 32-year-old woman consulted in October 2017 for an abscess on the upper external left thigh. Since the age of 2 years, she had been treated sequentially for juvenile rheumatoid arthritis with different disease-modifying antirheumatic drugs: methotrexate, sulfasalazine, leflunomide and, from 2001 to 2014, tumor necrosis factor inhibitors (etanercept, adalimumab, infliximab). She stopped them in 2014 to conceive. After her first pregnancy and uncomplicated delivery, she received tocilizumab from July 2015 to August 2017, then stopped for a second pregnancy. Afterwards, she took low-dose prednisone (5 mg/day) and ketoprofen.

She underwent bilateral hip arthroplasty, the right in 2005 and left in 2006, without any complications. She was well until October 2015, when sudden pain and swelling developed on the left external upper part of her thigh, without fever or rigors. Trochanteric bursitis was diagnosed on ultrasound. In November 2016, the bursitis was excised surgically and two wires were removed. Cultures of intraoperative samples remained sterile. No antibiotic therapy was prescribed. Two months later, swelling recurred and an abscess formed progressively. A draining sinus tract appeared in September 2017.

Physical examination in October 2017 found a productive sinus tract overlying the scar but no fever or hip pain, and was otherwise unremarkable. Her CRP was normal (<5 mg/L), white blood-cell count was elevated (20.55 G/L, with neutrophils at 17.67 G/L). X-rays of the left hip showed a cemented arthroplasty with acetabular and femoral loosening. Arthrography confirmed a communicating sinus tract through transfemoral osteolysis to the prosthesis. Bilateral hip aspirates were obtained; their Gram-stained smears were negative. Cultures (for methods see Case 1) of the left hip joint fluid grew *G. vaginalis*, identified by MALDI-TOF mass spectrometry (Bruker, Bremen, Germany; score 2.1), susceptible to penicillin, amoxicillin, clindamycin and rifampicin but metronidazole-resistant. No MICs were determined. The joint-fluid volume was insufficient to count leukocytes. Culture of the right hip joint fluid was sterile. Neither the patient nor her husband had a history of sexually transmitted diseases. Gynecologic examination was unremarkable. A vaginal smear and swab culture confirmed the presence of *G. vaginalis* with clue cells (Nugent score 7). Searches for other pathogens, and serology for antibodies to *T. pallidum*, human immunodeficiency virus and hepatitis B and C viruses were negative. Serum protein electrophoresis, immunoglobulins G, A and M, and complement levels were normal.

The patient underwent one-stage exchange arthroplasty on 12 December 2017. Two of the five intraoperative samples grew *G. vaginalis*. Postoperatively, she received continuous IV clindamycin (2400 mg/24 h) for 3 weeks, followed by an oral regimen (clindamycin 750 mg, TID) for 9 weeks, to complete 12 weeks of antibiotics.

One-year postsurgery she had no pain, the scar was unremarkable, CRP was 36 mg/L; persistent inflammation of her right knee and elbow was treated with steroids (prednisone 15 mg/day) and ketoprofen. X-ray of the hip showed no loosening, osteolysis or periosteal bone formation.

## Discussion

We describe two chronic PJIs due to *G. vaginalis*, an extremely rare infection. Indeed, only one case report has been published to date 10 and, during 30 years of management of these infections in our specialized Bone-and-Joint Infection Referral Center, we have observed only two cases among 2065 PJIs treated. One could speculate that modern identification techniques, such as MALDI-TOF mass spectrometry, could have contributed to diagnosing these infections more recently. Nevertheless, osteoarticular *G. vaginalis* infections remain exceptional 6-10.

In our patients, the bacterium also grew in our patients' vaginal swab cultures, with high Nugent scores, indicating BV, despite the patients' having no symptoms of genital tract infections. Case 1 was immunocompetent and postmenopausal, and Case 2 was a young immunocompromised woman. The long symptom-free intervals between the last clean operation and PJI signs, sudden onsets of PJI symptoms and identification of the portals of entry, strongly suggest hematogenous bacterial spread from the vagina to the prosthesis. Bacteremia was not confirmed, but both patients consulted months after the acute phase of PJI that had become chronic.

BV etiology and the precise role of *G. vaginalis* in it remain matters of debate 2-5. BV is a polymicrobial condition involving polymicrobial biofilm 4. *G. vaginalis* appears to be the predominant and most virulent BV-associated anaerobe, demonstrating marked adherence to the vaginal epithelium and biofilm-producing capacity 2. Biofilm formation on contraceptive intravaginal rings in African women was investigated recently 13, but studies on extravaginal biofilm formation are lacking. The rarity of *G. vaginalis* extravaginal infections and bacteremia suggest this bacterium's low pathogenicity. Bacteremia after gynecologic infections or delivery has been described 14, as were several urinary tract infections in men 15. Notably, that uncommonness and low pathogenicity could explain the rarity of this bacterium in PJIs, even though it is an efficient biofilm producer and, most certainly could do so even on prosthetic joints.

Five *G. vaginalis* bone-and-joint infections have been reported 6-10, including only one PJI 10 (Table 1). All developed in women, except parietal bone osteomyelitis in a newborn 6. Vaginosis and *Gardnerella* colonization, sought for three of the five cases, was positive only for the infant's mother. A late prosthetic hip infection developed 10 years after arthroplasty in an immunocompetent postmenopausal woman 10, suggestive of hematogenous spread, as for our cases. That woman presented with unilateral hip pain, fever, normal radiography, elevated CRP and sterile blood cultures. Cultures of joint fluid collected by hip aspiration were sterile. She underwent one-stage exchange arthroplasty and cultures of intraoperative samples isolated *G. vaginalis*. She had no signs of vaginosis and cultures of vaginal and urine specimens were sterile. Pertinently, as for our cases, the hip was involved. Although the number of *Gardnerella* PJIs is very low, the hip, close to the genital tract, could be at higher risk of contamination than the knee.

Treatment of our patients' chronic PJIs combined single-stage exchange arthroplasty and prolonged antibiotic therapy. In our experience 16 and that of others 17, a fistula is not a contraindication to one-stage exchange arthroplasty, as outcomes for these patients are not worse. We initially chose clindamycin rather than amoxicillin for its good activity and bone diffusion, but Case 1 developed an allergic rash and clindamycin was replaced by amoxicillin. Treatment lasted 12 weeks, with 3-4 weeks of IV therapy.

*G. vaginalis* is susceptible to various antibiotics: penicillin, amoxicillin, vancomycin, metronidazole and clindamycin. Quinolones and co-trimoxazole are less effective. Metronidazole is the treatment of choice for vaginosis. No consensus exists on the treatment of systemic infections. Among the five patients treated for bone-and-joint infections, three received amoxicillin or ampicillin 6-8, one patient each was prescribed clindamycin or co-trimoxazole 9, 10, and rifampin 8, 10 or quinolones 9 were added to three patients' regimens. Treatment lasted 5-8 weeks.

In conclusion, *G. vaginalis* is a very infrequent agent of PJI in women, even those postmenopausal. This biofilm-forming bacterium usually spreads hematogenously from the genital tract. Prolonged cultures on enriched medium and identification by MALDI-TOF mass spectrometry are important diagnostic tools. There seems to be no specificity for the surgical and medical treatment of these chronic PJIs, other than to search for a portal of entry by looking for prior gynecologic interventions or complications, performing a thorough gynecologic examination and obtaining vaginal smears. However, because these infections are extremely rare, routine vaginal screening for *Gardnerella* in female patients undergoing prosthetic hip arthroplasty does not seem to be indicated, except when the patient has BV symptoms or prior episodes of it. In that case, BV should be confirmed and treated before arthroplasty.

## Figures and Tables

**Table 1:** *Gardnerella vaginalis* bone-and-joint infections

Site (reference)	Sex/age; Other Circumstances	Samples Yielding *G. vaginalis*	Search for *G. vaginalis* in Vaginal Sample	Antibiotic Regimen (IV/total duration); Surgery	Outcome
Spondylitis L5S1 (7)	F, 50 years	Vertebral biopsy	ND	Ampicillin + sulbactam (6 weeks/NA)	Favorable
Spondylitis L2L3 (9)	F, 38 years	Vertebral biopsy	ND	Clindamycin + ciprofloxacin (10 days/8 weeks)	Favorable
Hip arthroplasty (10)	F, 71 years	Intraoperative	Negative	Piperacillin-tazobactam + vancomycin followed by co-trimoxazole + rifampicin (10 days/5 weeks); one-stage exchange arthroplasty	Favorable
Hip arthritis (8)	F, 48 years; kidney transplant	Intraoperative	Negative	Amoxicillin + pefloxacin + rifampicin (2 weeks/6 weeks); hip arthroplasty	Favorable
Parietal osteomyelitis (6)	Newborn	Placenta, fetal side	Mother's sample positive	Amoxicillin (4 weeks/6 weeks)	Favorable
Hip arthroplasty (this study)	F, 68 years; postmenopausal F, 32 years; DMARD- treated juvenile rheumatoid arthritis	Hip aspiration, intraoperative samples	Positive for both patients	Clindamycin IV (10 days), switched to amoxicillin (4 weeks/12 weeks) Clindamycin (3 weeks/12 weeks); one-stage arthroplasty for both patients	Favorable for both

IV, intravenous; F: female; ND: not done; NA: not applicable. DMARD: disease-modifying antirheumatic drugs.
